# Toward the crystallographic and microstructural mechanisms of plant leaf waxes as diffusion barriers

**DOI:** 10.1039/d5ce01041a

**Published:** 2026-02-09

**Authors:** Sean M. Collins, Neil George, Andy Brown

**Affiliations:** a Department of Materials, Imperial College London, Royal School of Mines Exhibition Road London SW7 2AZ UK s.m.collins@imperial.ac.uk; b Retired Scientist Leeds UK; c School of Chemical and Process Engineering, University of Leeds Woodhouse Lane Leeds LS2 9JT UK

## Abstract

Waxes within the leaf cuticle, the outermost layer of the plant leaf, play a defining role as a transpiration barrier and also serve as an important target for agrochemical interventions for crop protection. A prevailing model for this behaviour is the role of wax ‘bricks’ in building the diffusion barrier. This review brings together crystallographic and microstructural research to highlight the variety of crystalline, disordered, and amorphous structural features known in waxes. We trace two predominant research routes applied to leaf waxes: one directed at simplified waxes but with highly detailed descriptions of molecular packing and a second focused on the diffusion characteristics of the complex system of the cuticle and its multicomponent wax compositions. Bringing these routes together will develop sufficiently complex but tractable structural models for waxes, often dominated by a single or a few components in common crop plants. A complete description of leaf wax function will enable ways to control diffusion through the development of targeted interventions for drought tolerance.

## Abstract 2

Waxes in the leaf cuticle act as a barrier to water loss as well as to agrochemicals. The structural origins in the varied but remarkably consistent (dis)ordering of long-chain hydrocarbons holds the key to refining mechanisms to control properties.

## Graphical abstract

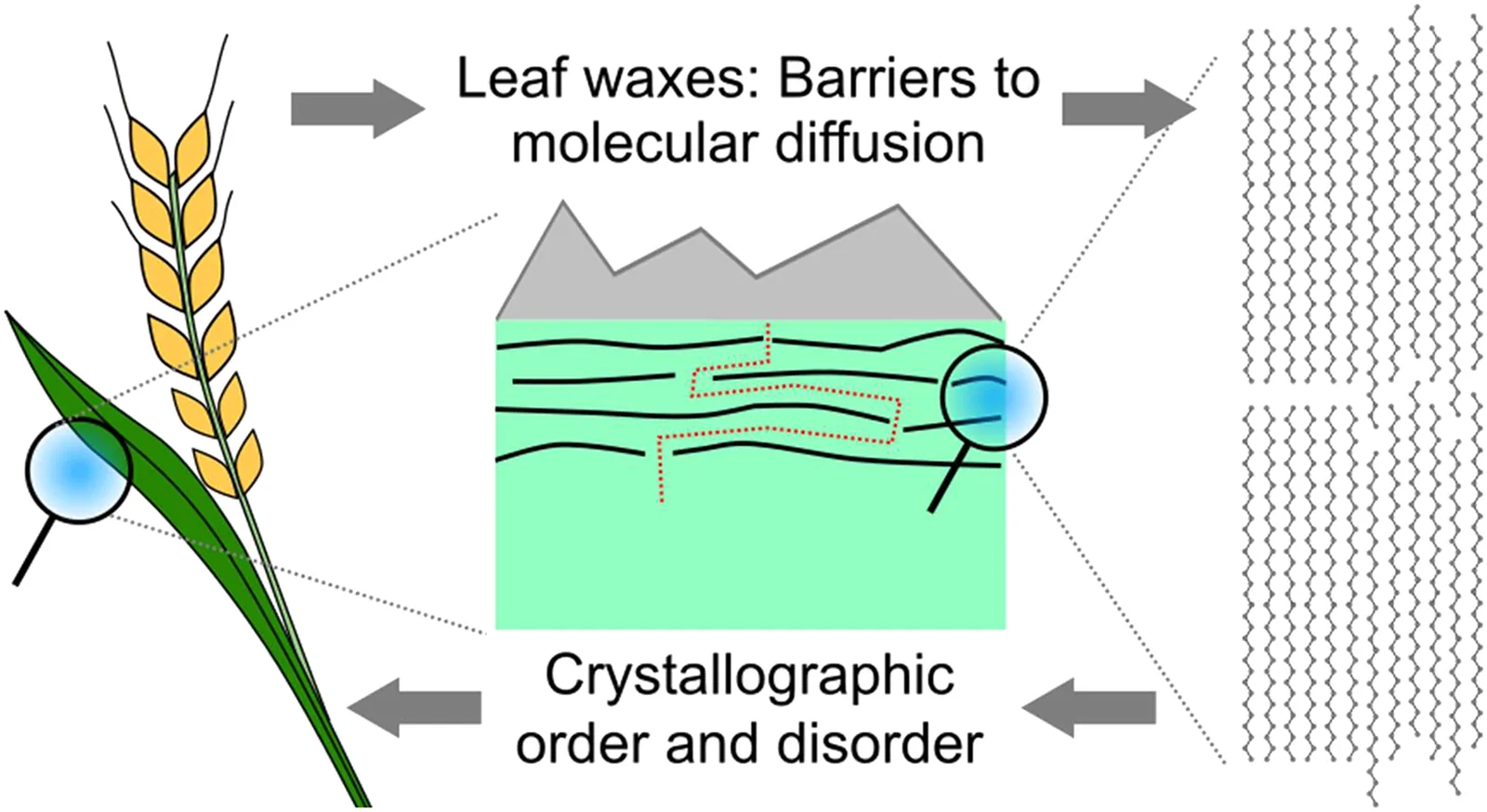

## 1. Introduction

Plant leaf waxes serve a host of critical functions, most notably acting to prevent water loss but also contributing to mechanical integrity, UV protection, and modifying the surface wettability of plants.^1–6^ Appearing in multiple layers at the surfaces of plant leaves termed the cuticle, leaf waxes are an important semicrystalline biomaterial. The functional properties of leaf waxes, such as acting as a barrier to water loss, are likely controlled by the plant through directed molecular synthesis and solid state assembly to control diffusion. Advances in crystallographic and microstructural understanding of leaf waxes are therefore needed given the evidence that diffusion in organic solids is governed by tortuous paths through mixed crystalline, disordered, and amorphous phases.^7,8^ Progress in understanding leaf waxes is particularly pressing in the face of global climate change and its particular consequences for food security and crop health,^9^ motivating crystal engineering solutions for resistance to pests and drought tolerance.

This review highlights the state of understanding of the crystalline structure of leaf waxes, including identifying sources of disorder and how this structural description translates to functional properties. We note a number of prior reviews have considered the crystallinity of the outermost wax layers termed the epicuticular waxes^10,11^ as well as plant surface structures and their interactions with the environment^2,12^ and the properties and functions of the leaf cuticle.^13^ The interplay between amorphous and crystalline structures is a well-established concept in leaf waxes.^14^ We turn our attention in this review specifically to the key question of disorder within semicrystalline waxes, with a particular focus on the crystallographic features relevant to waxes within the leaf cuticle (intracuticular waxes) at the critical length scales of molecular packing and nanoscale organisation which remain far from completely described. We posit that moving beyond simple descriptions of amorphous and crystalline fractions is needed, with the aim to shift toward a more rigorous characterisation of the microscopic organisation and atomistic models for the crystalline and non-crystalline domains in leaf waxes, *i.e.* the microstructure. In doing so, the multiple possible structural origins of increased or decreased tortuosity in the wax (*e.g.* size, orientation, phase fraction, and disorder modes), will be deconvolved. Achieving accurate microstructure–function relationships will identify the specific, active structures controlling diffusion to produce an understanding of microscopic mechanisms needed for designing more efficacious and targeted interventions for the protection of crops (by optimal exploitation of the mechanism in the delivery of active ingredients and adjuvants) as well as for enhancing drought tolerance beyond crops in the conservation of ecologically important plants.

The leaf, however, is a compositionally complex system containing waxes (often comprising multiple long-chain hydrocarbons), polymeric and polysaccharide components, and small molecules interacting with the cells and other, extracellular structures of the plant. Composition is further complicated by variations between species, location, environment, and plant development. Analyses of crystalline structure of waxes either tend toward simplified structures comprising a single component or a few components or tend toward a complex mixture extracted and separated from the biological context of the leaf. This tension arises from the practicalities of analytical techniques and conceptual constraints for interpretation of such systems.

**Fig. 1:**
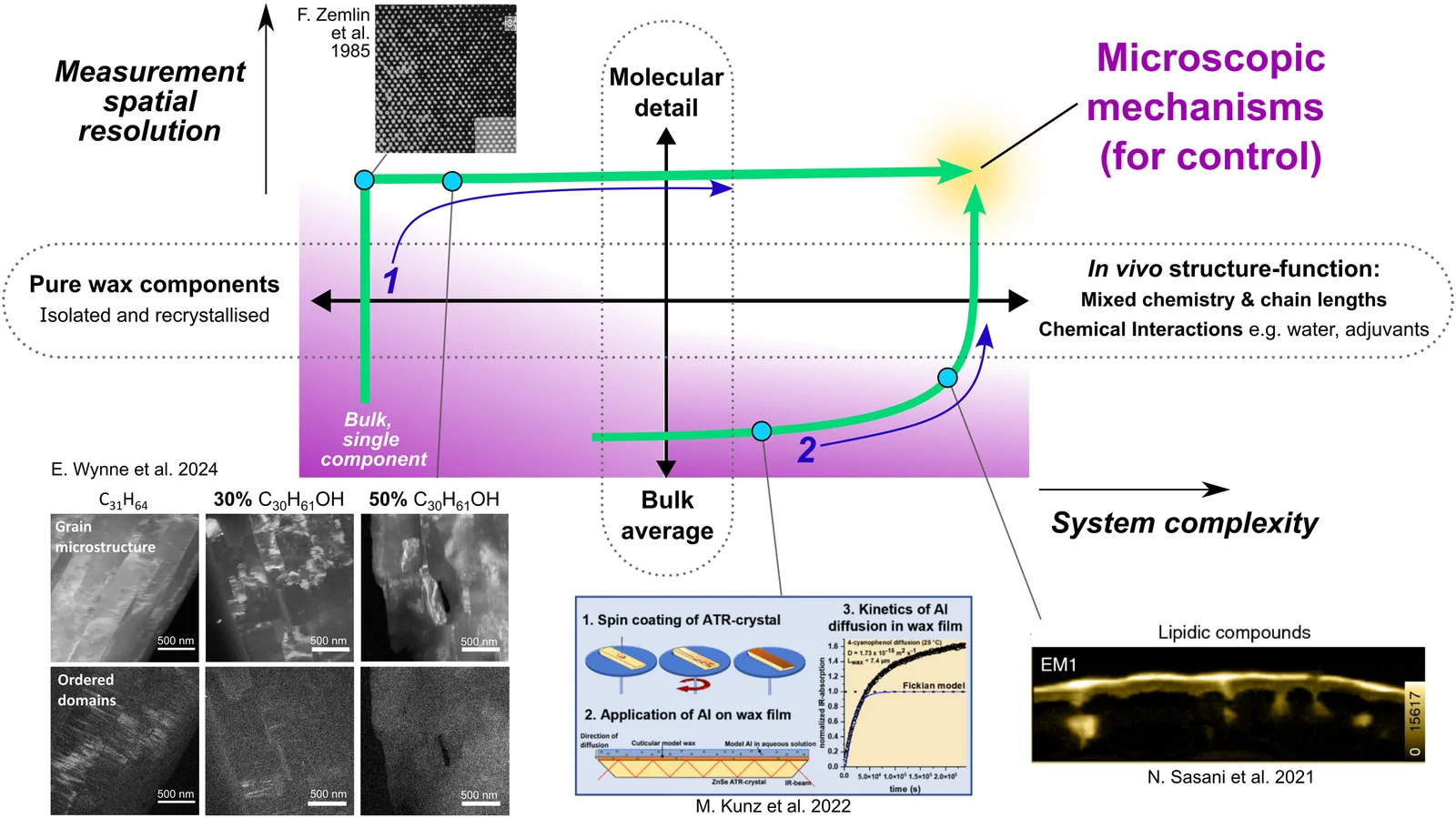
Schematic overview of the progression toward microscopic mechanisms underpinning structure–function relationships in leaf waxes. Two principal routes (dark blue labels) are identified: (1) by way of detailed analysis of purified and simplified systems, supporting molecular detail at high spatial resolution and (2) through bulk analyses of complex systems offering links to macroscopic properties. Complete mechanistic understanding depends on bringing these two routes together. The schematic incorporates elements reproduced from ref. 15–18. F. Zemlin *et al.* 1985 (ref. 15) reused with permission (copyright 1985, The American Association for the Advancement of Science). M. Kunz *et al.* 2022 (ref. 16) reused with permission (copyright 2022, The American Chemical Society). E. Wynne *et al.* 2024 (ref. 18) and N. Sasani *et al.* 2021 (ref. 17) reused with permission (CC-BY 4.0).

This divide between simple and complex systems establishes two main routes for arriving at microscopic mechanisms that we also trace through this review (Fig. 1): (1) a route focused on isolating pure or simplified components for analyses of molecular packing and conformational details (nanometre to atomic scale) and (2) a route focusing on macroscopic properties of complex mixtures making use of bulk structure averaged over the length scales much larger than the molecule or unit cell dimensions. In turn, both increasing the complexity of systems studied at high spatial resolution (route 1) and increasing the spatially resolved and atomistic detail of descriptions in complex, multi-component and *in vivo* function (route 2) are key paths forward to unravel the microscopic mechanisms that underpin the vital diffusion barriers created by leaf waxes. We note that an ideal model will likely offer an elegant description based on only a few defining wax components and their crystallographic features; nevertheless, for validation it must be assessed against the full complexity of the leaf cuticle.

We organise this review first by providing a brief overview of the structure and chemistry of the plant cuticle and the prevailing structural descriptions. We then follow routes 1 and 2 through the literature, first reviewing unit cell descriptions of the principal long-chain hydrocarbon components and their disorder modes and then turning to the identification of the defining role of intracuticular waxes in creating diffusion barriers and the modes of action proposed for agrochemical adjuvants. We then conclude with perspectives on unresolved questions and key directions for future work.

## 2. Overview of the plant leaf cuticle

We briefly introduce the leaf cuticle here to establish the compositional and biological context for understanding crystalline ordered and disordered structures in long-chain hydrocarbon waxes. The biology of the leaf cuticle has been reviewed previously^2,4,12,14,19–22^ and is not our main focus here; rather, we seek to outline key principles and reference points for considering wax microstructure.

**Fig. 2:**
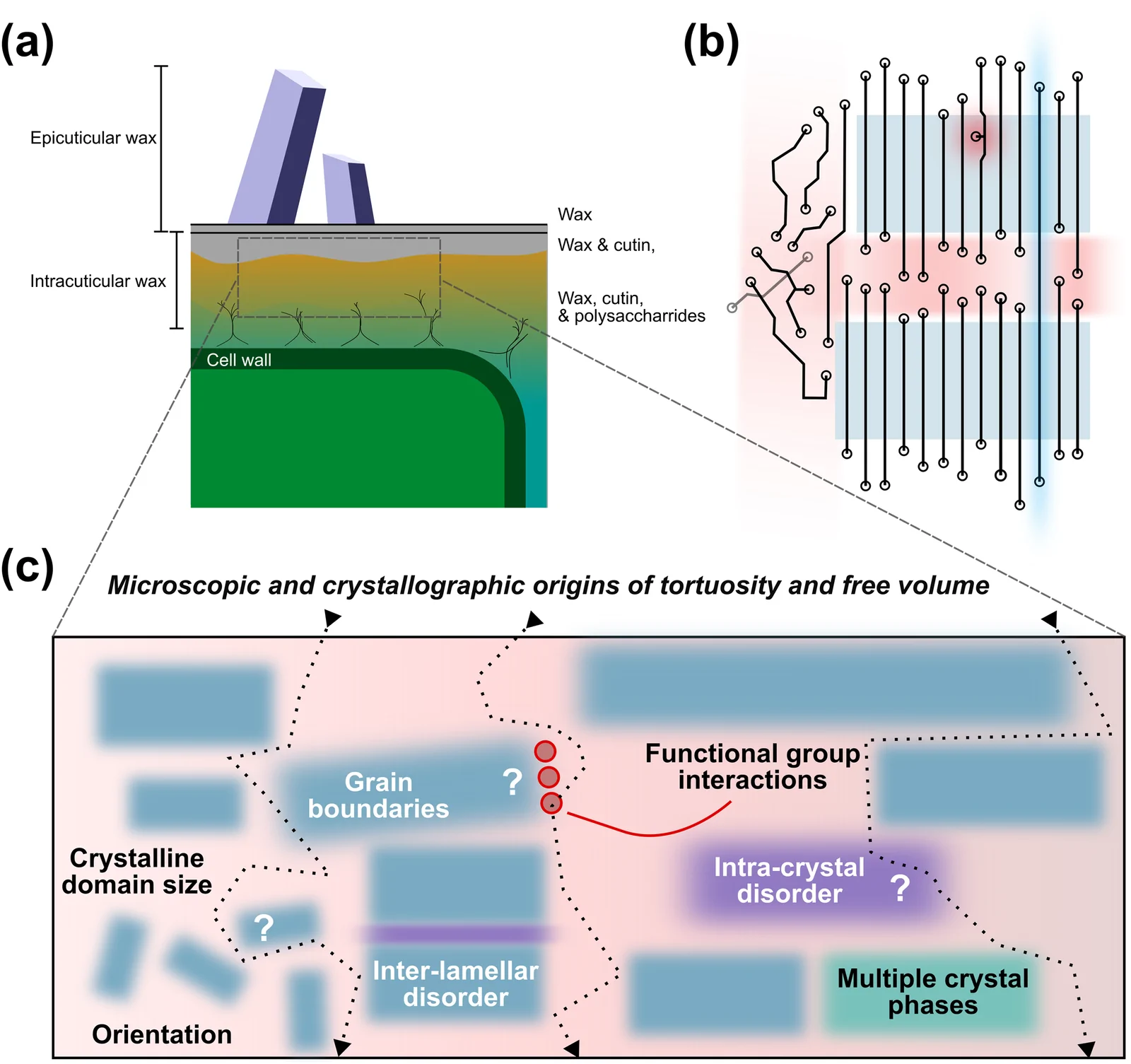
(a) Diagram of the epicuticular and intracuticular wax layers on the surface of plant leaves. (b) The prevailing structural description used to describe the general crystalline and amorphous features of leaf waxes. Regions in red have been described as amorphous regions, and regions in blue depict crystalline structures with defined molecular packing between aligned long-chain molecules within polyethylene-like block-like regions or lamellae. (c) An illustration of the barrier-membrane model of the intracuticular region showing crystalline ‘bricks’ (blue, green, and purple) in an amorphous matrix (pink). Molecules are considered to diffuse predominantly in the amorphous matrix. The relative fractions of crystalline and amorphous phases drawn are not intended to reflect a particular ratio but are presented for visual clarity. Pathways of molecular species diffusing through the cuticle are indicated by dashed lines, highlighting changes in diffusion (marked by question marks) due to changes in packing density even at fixed phase fractions, different chain end functional group interactions, or different degrees of intra-crystal disorder. Refinement of such models beyond quantifying the crystalline or amorphous fraction requires assessment of crystal domain size, orientation, functional group packing in the solid state, different crystalline phases present, and an atomistic description of disorder within crystals (including disorder between lamellae).

The cuticle comprises a set of layers on the surface of leaves outside of the cellular structures. Fig. 2a presents a schematic overview noting the positions of cells bounded by their cell walls covered, in closest proximity, by a mixture of wax, cutin, and polysaccharides.^14,20,23^ Waxes refer to the discrete long-chain hydrocarbons (including long-chain alkanes, alcohols, fatty acids, esters and aldehydes) whereas cutin refers to a polymeric matrix (a polyester derived from fatty acids). Polysaccharide fibres form a bridging region from the epidermal cells, sometimes referenced as the pectin layer^14,24^ and distinguished with the region immediately above it, termed the cuticular layer with reduced polysaccharide content. Near the surface of the leaf, the layers become predominantly wax and cutin, termed the cuticle proper.^25^ The top-most layer, located above the intracuticular wax, is the epicuticular wax layer. This layer consists of wax only and exhibits the greatest degree of apparent crystallisation, with faceted crystals (grains, plates), filaments or tubes of wax characteristically visible on the surfaces of many plant species.^10,11,25,26^

Compositionally, cutin comprises lipid polyesters resulting from the esterification between alcohol and carboxylic acid moieties on saturated, multiply hydroxylated aliphatic acid monomers.^14,27^ Here and throughout, we denote a chain length with *n* carbon atoms as C_*n*_. In cutin, the monomers typically consist of C_16_ and C_18_ carbon chains.^14,27^ The wax components tend to be long-chain hydrocarbons (typically >C_20_), spanning a range of functional group chemistries dominated by alkanes, primary alcohols, fatty acids (sometimes referred to as very long chain fatty acids, VLCFAs), as well as aldehydes, alkyl esters, and secondary alcohols, ketones, and related chemistries in specific plants.^20,28^ Triterpenoids, phenolic, and other compounds may also be present, though typically as minor fractions in leaf waxes.^14,28^

The long-chain hydrocarbons in leaf waxes also originate from biosynthesis of the C_16_ and C_18_ fatty acids, however, wax synthesis occurs within the epidermal plant cells.^20,29,30^ The biosynthetic pathway involves elongation of the fatty acid to *n* ∼ 26–30 (even *n*) or longer followed by a divergence along two main routes: (a) the acyl reduction or reductive pathway and (b) the decarbonylation or decarboxylation pathway.^14,20,30^ The acyl reduction pathway produces even-*n* C_*n*_ primary alcohols (*n*-alkanols) whereas the decarbonylation pathway produces odd-*n* C_*n*_ normal alkanes (*n*-alkanes) through an even-*n* fatty aldehyde intermediate. These alcohol and alkane pathways have been traced in particular species to identify the critical reductase^31^ and decarbonylase^32,33^ enzymes. This tendency to form odd-*n* alkanes and even-*n* alkanols has specific implications for considering crystalline packing (see section 3 below). Notably, cutin is synthesized outside of the cell, whereas wax molecules are synthesized in the cell and transferred to the outer leaf surface, thereby building the waxy regions from the bottom up. This route necessarily involves the wax molecules passing through the plasma membrane, polysaccharides, and cutin to reach the intracuticular or epicuticular wax layers, from polar and hydrophilic to increasingly nonpolar regions.

The multi-layer model of the leaf cuticle (Fig. 2a) has emerged from studies of the composition of leaves, revealing differences in the relative fractions of major and minor components across the epicuticular and intracuticular wax layers.^34,35^ Typically, either solvents, collodion-based removal, mechanical wiping, cryo-adhesives, or polymer adhesives have been used to mechanically remove the epicuticular wax layer for subsequent extraction and compositional analysis by gas chromatography and mass spectrometry.^35^ The remaining cuticular wax can be extracted by solvation, *e.g.* in chloroform.^34^ These analyses have been particularly important where the epicuticular layer does not exhibit a distinctive, particulate morphology but nevertheless remains a distinguishable feature of the leaf surface seen by compositional differences. Fernández *et al.* have questioned the rigid distinction between layers within the intracuticular region and raised the potential significant role of polysaccharides and the interactions with the cell wall as important features in considering leaf surface, properties, and stress response characteristics.^36^ In any case, the conceptual division among regions of differing chemical composition, however sharp or diffuse, remains useful in delineating the epicuticular and intracuticular regions principally where these also serve discrete functions.

Significantly, the intracuticular waxes have been shown to be the essential feature for transpiration control (see sec. 4),^5,6^ acting as the crucial barrier (albeit not a perfectly impermeable one) to water loss from the surface of leaves and allowing the plant to control water content in the leaf effectively through the stomata.^19,37^ In some species like *Schefflera elegantissima*, the long-chain *n*-alkanes dominate the intracuticular wax,^5^ pointing to the alkanes as a prominent feature underpinning transpiration control at least in some plants and underscoring the possibility that simplified compositional models may be suitable to explain a significant amount of leaf wax function.

The epicuticular waxes play key roles as the outermost layers, particularly prominent in determining the surface wettability and interactions with water droplets on the surface, adhesion by solid particles including fungal spores, pollen, or soil, interactions with insects, and also contribute to UV protection.^1,3,12^ The cuticle as a whole likely contributes to the mechanical properties of the plant, with a reported Young's modulus (elastic modulus) of isolated cuticles between 0.6–1.3 GPa,^3,38^ matching or exceeding values for isolated polymers like polyethylene (0.6–0.8 GPa).^39^ Moreover, these properties appear to vary with hydration, with a reduction in stiffness observed with increasing hydration, suggesting a plasticising effect of water on the mechanical properties of the cuticle.^38^

In order to offer a general explanation of the relationship between structure and these key functional properties of leaf waxes, a description of waxes as semicrystalline materials has been advanced.^14,40–43^ This model (Fig. 2b) consists of three main regions: (i) there are ordered regions with molecules (lines) arranged in parallel; (ii) there are disordered regions in between these ordered blocks arising from different molecular lengths or terminal group conformations; and (iii) there are further regions, likely at the edges or interstices of the ordered material, with no long range order and containing a range of chain orientations and conformations. Additional individual molecule features arising from branched alkyl chains or functional groups (*e.g.* secondary alcohols or ketones) may introduce deviations from perfect order within ordered regions. Conversely, very long chains may bridge between ordered regions introducing an additional degree of ordering between adjacent blocks of aligned (parallel) chains.

The aligned molecular block regions have been referred to as crystalline, and the regions in between the blocks and adjacent to the blocks have been referred to as amorphous. Often those regions termed amorphous have been subdivided further into solid amorphous and mobile amorphous phases.^43^ From a crystallographic perspective, these terms are overly broad. In particular, the solid amorphous designation, often referencing the disordered chain-end regions between lamellae, would be more correctly identified as crystalline given that this is well-described with reference to the unit cell; where these regions still exhibit side-by-side chain packing they are sufficiently ordered to be expected to exhibit Bragg diffraction. We refine these descriptions in sec. 3.

This model has been developed mainly from nuclear magnetic resonance spectroscopy studies of extracted leaf waxes (and also Fischer–Tropsch waxes) indicating predominant alignment of the molecular long-axis in an all-*trans* zig-zag conformation.^40–43^ Additional signals have been assigned to regions with greater conformational freedom (*e.g.* under thermal activation) for end-group *gauche* conformational changes.^40,41,43^ Additional insights from selected area electron diffraction (100 keV electron beam energy) have confirmed the presence of ordered lamellae or lamellar domains of aligned long-chain alkanes in plant waxes (Brazilian palm leaf wax) and insect waxes (beeswax)^44^ as well as bridging between lamellae in synthetic waxes (C_60_–C_68_ and C_34_–C_36_).^45^

Whilst much of this conceptual model development has arisen from extracted and recrystallised leaf waxes or petrochemical sources, it has been established from early work that waxes scraped from plant surfaces exhibit X-ray diffraction signals corresponding to end-to-end chain packing of wax components,^46^ confirming crystallinity at least of the epicuticular waxes. However, it should be noted that Fourier transform infrared (FTIR) spectroscopy studies^47^ have also confirmed a fractional degree of crystallinity in whole leaf studies (as estimated from characteristic spectroscopic signatures) benchmarked against isolated cuticular membranes and extracted wax samples in plant leaf samples with high cuticular wax content (to minimise interference otherwise from the cutin polymer).^48^ FTIR spectroscopy has further enabled investigation of the configurations of water within ivy leaves, informing models of water interactions with carbonyl moieties in cutin.^49^

NMR and FTIR spectroscopies are especially valuable for assessing molecular conformations in both crystalline and amorphous phases as they are not crystallographic probes. At the same time, attribution of crystallinity from ratios of conformations or relaxation times may require some care. For example, *gauche* conformations can also occur within disordered but crystalline molecular packing (section 3). We note that NMR studies have suggested 52% of extracted wax (after reconstitution) from barley (*Hordeum vulgare)* leaves is crystalline.^43^ FTIR studies within leaves of *Hedera helix* and *Juglans regia* suggest crystallinity fractions of 74% and 58%, respectively.^48^

This conceptual model for leaf waxes as semicrystalline materials has underpinned the prevailing description of permeability and diffusion of species through leaf waxes in terms of a barrier-membrane model (Fig. 2c),^14^ in analogy to barrier membranes (*e.g.* polymer–mica) where diffusion occurs only in the amorphous regions between ‘bricks’ (as in a brick wall) that are presumed to be perfectly impermeable.^7^ In turn, this establishes a principle that the path for diffusion occurs only in the high-mobility ‘amorphous’ fraction of leaf waxes, limiting the volume for diffusing molecules as well as introducing tortuosity.^14^ This model has sometimes been described as a percolating network through a crystalline matrix, a description that is suggested by estimates of a high crystalline fraction.^50^ Notably, models have typically assumed a ‘limiting skin’ in the cuticle,^51^ referring to a relatively thin section of the cuticle rich in wax phases with low-mobilities for water or other diffusing species. As such, the average fraction of low-mobility, ordered or partially ordered crystalline waxes in the leaf is not as important as the number of ‘bricks’ and their lateral extension, restricting the higher permeability phases (comprising amorphous wax and cutin) to small gaps and tortuous paths.^51^ We focus our attention on the crystalline ‘bricks’ as the size and packing of these structures is believed to control molecular diffusion. In turn, an improved description of the ‘bricks’ will define the quantity, location, and distribution of the amorphous phases, though we recognise their structure and properties undoubtedly warrant further investigation as well. Within the barrier membrane model, however, the key feature is not the identity of the amorphous, high-mobility phase (provided it exhibits higher mobility for diffusing species) but rather the ordered or partially ordered molecular packing in crystals and their size that comprise the low-mobility phase disrupting and slowing diffusion.

This barrier membrane picture has gained traction in the experimental work on extracted and reconstituted leaf waxes. By measuring diffusion coefficients of a series of radiolabelled compounds in reconstituted leaf waxes, Kirsch *et al.* demonstrated that these values could be used to reliably predict the permeance of the same compounds in leaves and isolated cuticles.^52^ Attempts to replicate leaf wax compositions with matched ratios of purified hydrocarbons, however, have only partially replicated phase transition behaviour of the more complex leaf wax compositions.^42^ Nevertheless, self-assembly of extracted waxes reproduces crystal morphologies observed on leaf surfaces,^53–56^ supporting the use of reconstituted waxes as models for their forms in the leaf. Taken together, extracted and isolated leaf waxes raise the possibility of explaining important structural features as well as functional properties through the study of simplified and replica leaf waxes; we now turn to the structure of such waxes in sec. 3.

Inferring key structure–function relationships from simplified and replica leaf waxes should be done with care to avoid over-generalising from single- or few-component wax mixture models without considering the correspondence to functional response of *in vivo* compositions and structures. However, there is cause for optimism for understanding major crops leaf waxes as many cereals show a predominant contribution from a limited number of long-chain hydrocarbons: Leaf waxes in wheat are ∼66% 1-octacosanol (C_28_H_57_OH),^56,57^ in barley are ∼80% 1-hexacosanol (C_26_H_53_OH),^42,58^ in maize are over 40% alkyl esters (C_44_ and C_46_ especially),^59,60^ and in rice are over 40% 1-triacontanol (C_30_H_61_OH).^61,62^ While outsized effects from minor components cannot be ruled out, the structural features and linked properties are expected to follow the structural and chemical properties of the single or few-component mixtures of the major wax constituents. As we now show in sec. 3, molecular packing in waxes of highly varied compositions exhibits a substantial degree of commonality which aligns with the barrier membrane model in explaining a consistent formation of low-mobility crystalline regions in diverse cuticles.

## 3. Route 1: order and disorder in simplified waxes

In ordered form, long-chain hydrocarbons pack side-by-side in parallel strands (as illustrated in Fig. 2). However, this general structural motif leaves a range of possibilities for the specific molecular packing in a crystal structure. In this section, we start by introducing unit cell descriptions of single-component waxes (*i.e.* single chain length and functional group) before discussing mixtures and disorder modes, referenced relative to their deviations from the single-component unit cells. The crystallographic description of long chain hydrocarbons encompasses far more than leaf waxes, with significant intersection with petrochemical waxes, coatings, and other wax-derived chemical products as well as with related polymer structures. We focus this review on leaf waxes, drawing on comparisons or literature on these related systems where they grant further insight into leaf wax structures.

Firstly, the molecular structure detail beyond a linear chain is a defining characteristic of waxes. Comprising sp^3^ carbons, the long-chain component of waxes consists of methylene groups (R–CH_2_–R). Adjacent methylene groups characteristically adopt a conformation where the smaller protons are brought closer together while minimising steric clash of the carbon–carbon chains, *i.e.* a *trans* conformation. In the extended form, this results in a zigzag of the carbon atoms along the chain, better described by a repeating ethylene unit (R–CH_2_–CH_2_–R). This structural motif is identical to the repeat in polyethylene (PE) polymers, with the distinction that a single-component wax comprises a single chain-length (C_*n*_), typically on the order of *n* ∼ 21–35 carbon atoms for *n*-alkanes in leaf waxes. For comparison, heneicosane (C_21_H_44_) has a molecular weight of ∼3 × 10^2^ g mol^−1^ whereas PE polymers tend to have molecular weights in excess of 10^4^ g mol^−1^.

**Fig. 3:**
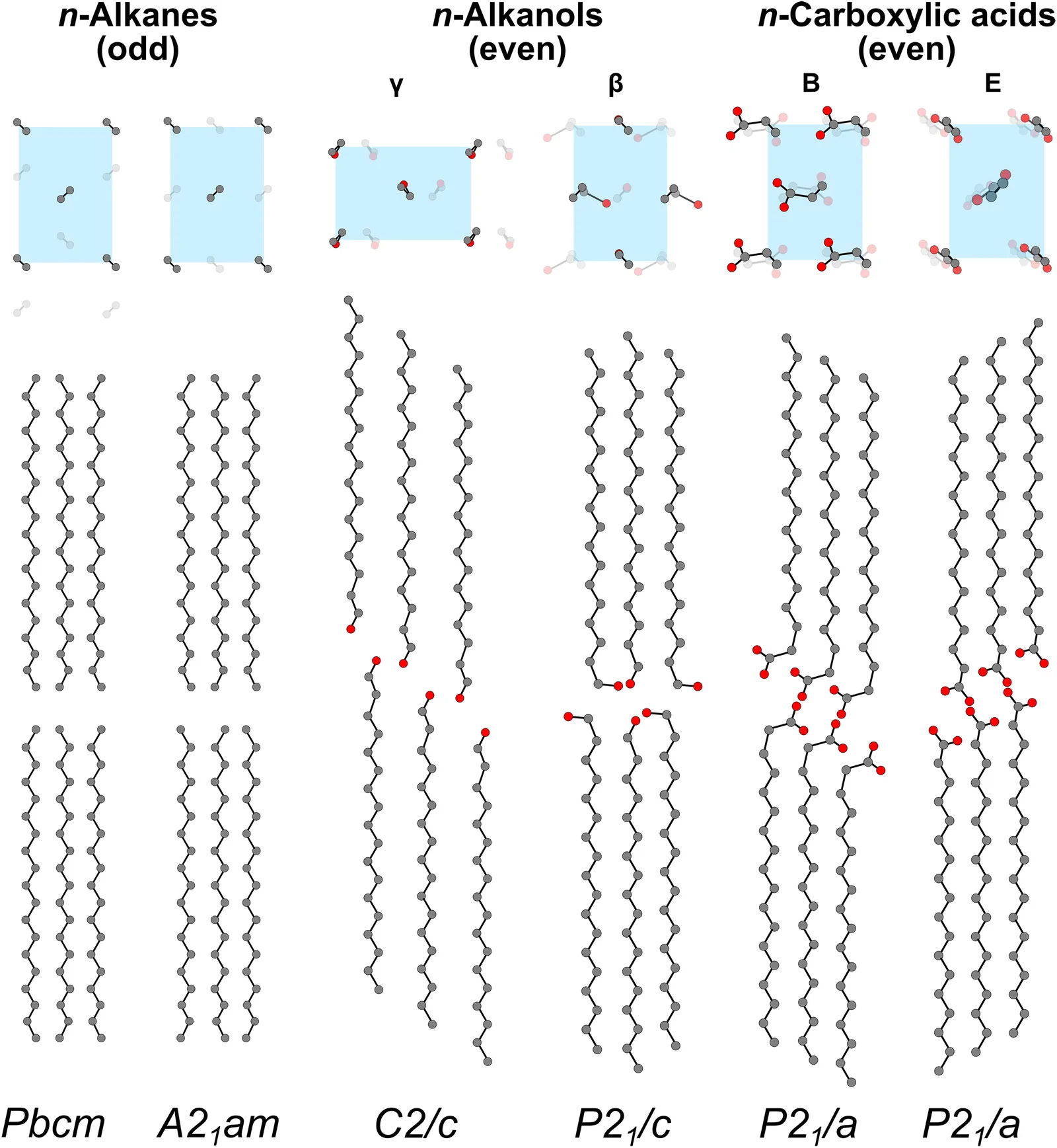
Diagrams depicting the molecular packing arrangement in reported unit cells for odd-*n* long-chain alkanes (*n*-alkanes), even-*n* long chain primary alcohols (*n*-alkanols), and even-*n* fatty acids (*n*-carboxylic acids). The molecules are shown viewed along the long-axis of the molecule (‘down-chain’) at the top and viewed side-on below. A blue box indicates the polyethylene-like unit cell (sub-cell) for each structure. The space groups of each structure are given at the bottom. Carbon atoms are shown in grey, and oxygen atoms are shown in red. Hydrogen atoms are omitted for visual simplicity. The chain lengths shown (*n* = 18–19) are shortened for visual clarity relative to the longer chains present in leaf waxes (*n* ∼ 21–35 for *n*-alkanes).

Nevertheless, crystalline ordering of PE is a common reference point for waxes. The PE unit cell^63,64^ considers the structure to comprise infinitely extended chains, and so it establishes a cornerstone for chain-to-chain packing that is common to long-chain but finite-length hydrocarbons. The PE unit cell is orthorhombic, with the PE chains running along the *c*-axis. The cell can be described in terms of one ethylene unit positioned along the four unit cell edges (equivalent by translation) and a second ethylene unit positioned at the centre. The zigzag ethylene unit along a diagonal in the *ab*-plane therefore marks the nearest chain-to-chain interactions. In the PE unit cell, the plane containing the all-*trans* zigzag of one ethylene unit is approximately 90° from the all-*trans* zigzag plane of the next ethylene unit, establishing alternation of nearest-neighbour ethylene units in the PE unit cell. This characteristic arrangement of the *ab*-plane (viewed along the PE *c*-axis) is highlighted (blue shaded rectangle) in Fig. 3 for all depicted typical *n*-alkane, *n*-alkanol, and fatty acids relevant to leaf waxes.

Despite this effectively universal arrangement in the chain-to-chain interactions along the all-*trans* ethylene repeats in waxes, differences in chain packing arise in finite-length hydrocarbon chains due to the chain ends. That is, whereas the PE cell assumes infinitely long ethylene repeats, wax molecules are of defined length, and the terminal methyl or other functional groups introduce an important deviation from the PE unit cell. While many of the carbons in the backbone of long-chain hydrocarbons may readily adopt an ethylene repeat, at least two broad effects can be described emerging from the chain ends. First, the finite chain length means that molecules are packed end-to-end, and how these chains are stacked adds a further consideration in the overall three-dimensional packing. Second, the terminal functional groups or conformational changes may further modify the end-to-end intermolecular interactions significantly, *e.g.* through hydrogen bonding.

Following their biosynthesis (sec. 2), we focus here on *n*-alkanes with an odd number of carbon atoms, *n*-alkanols with an even number of carbon atoms, and *n*-carboxylic acids with an even number of carbon atoms (Fig. 3) to draw out illustrative examples as these reflect major constituents of common leaf waxes. Each of these molecules consist of an extended ethylene chain. Alkanes are terminated symmetrically with methyl groups whereas the *n*-alkanols and *n*-carboxylic acids contain a primarily alcohol or a carboxylic acid group at one end. Critically, odd-carbon *n*-alkanes and even-carbon *n*-alkanols (as well as even-carbon fatty acids) represent the major fraction in many intracuticular waxes, including coffee plants (*Coffea arabica*, dominated by acids, alcohols, and alkanes) and some arrowroots (*Calathea lutea)*^14^ as well as *Schefflera elegantissima* (dominated by alkanes and alkanols)^5^ and staple cereals like barley, wheat, and rice (dominated by alkanols).^42,56–59,61^ As such, these chemistries represent a useful minimal system for considering essential leaf wax components and for elaborating the different effects of terminal polar functional groups on the crystal structure of waxes.

Taking the *n*-alkanes with an odd number of carbon atoms first, Fig. 3 shows the commonly reported unit cell with *Pbcm* space group symmetry. The crystal structure has been determined from single-crystal measurements on tricosane (C_23_H_48_)^65^ and the space group symmetry has also been established across *n*-alkanes (C_*n*_H_2*n*+2_ for odd *n* = 13–41) by structure prediction^66^ and synchrotron powder diffraction measurements.^67^ Views along the ethylene chains (top) highlight the PE-like arrangement. The chains are packed side-by-side in layers or lamellar blocks with a gap between the lamellae. This stacking is visualised side-on (bottom). In this orthorhombic cell, there are two orthogonal high-symmetry side-on views. The side-on view shown is viewed along the longer edge in the top view. This view shows how the ethylene chains outline a seemingly approximately hexagonal arrangement of carbons. This graphite-like arrangement appears only in projection as the molecules are not co-planar in the three-dimensional cell. Here, the mirror symmetry with planes at the mid-point of the chain is apparent for odd-carbon *n*-alkanes. That is, a plane drawn at the centre of the chain can be used to reflect all atoms in the upper half onto the lower half (and *vice versa*).

A second polytype for odd-carbon *n*-alkanes has been observed in electron diffraction with orthogonal unit cell with *A*2_1_*am* space group symmetry in experiments led by Douglas Dorset,^68–70^ also identified as similar to the form of tritriacontane (C_33_H_68_) after a first phase transition (from the room temperature structure) at 54.5 °C (ref. 71 and 72) and resembling a similar crystal structure observed in solid solutions of even-carbon *n*-alkanes by X-ray diffraction.^73^ The thermally induced phase transitions in *n*-alkanes are largely beyond the scope of this work, but broadly consist of several solid–solid transitions including a rotator phase transition prior to melting, as reviewed previously.^74^ Interestingly, Dorset has reported the *A*2_1_*am* structure at room temperature in crystals recrystallised from the melt using a benzoic acid templating process for orientation control. We have separately reproduced these results,^18^ but whether this polytype occurs as a result of templating on benzoic acid or due to incomplete solid–solid phase transformations on cooling from the melt remains unresolved. Many of the intermediate elevated temperature phases have been described as twinned and defect-rich,^71^ and, in fact, the structural similarities between the *Pbcm* and *A*2_1_*am* structures (Fig. 3) largely consist of small changes in the offset between layers of otherwise aligned and similarly oriented ethylene chains. This type of polytypism hints at disorder modes between the ordered PE-like lamellae.

Compared to the *n*-alkanes, much more pronounced changes are introduced in the *n*-alkanol^75–81^ and *n*-carboxylic acid^82–91^ crystal structures. In terms of symmetry, the long-chain hydrocarbons no longer contain a mirror plane or inversion centre at the mid-point of the molecule; such a change in symmetry still allows for a range of orderings. With the incorporation of polar terminal groups capable of participating in hydrogen bonding, the landscape of intermolecular interactions is significantly altered, and strong hydrogen bonding networks are preferred. These chemical interactions result in unit cells with paired, end-to-end hydrocarbon chains to enable the formation of hydrogen bonding networks between every-other layer of long-chain hydrocarbons. Notably, the PE subcell is preserved, given that the side-to-side interactions remain relatively unaltered.

For the even-carbon *n*-alkanols, at least two polymorphs have been observed at ambient temperature (low-temperature forms), denoted γ and β.^76,79^Fig. 3 depicts the γ-form (monoclinic space group *C*2/*c*) following Michaud *et al.*^77^ and the β-form (monoclinic space group *P*2_1_/*c*) based on the β-heptadecanol crystal structure reported by Seto.^76^ We emphasise the molecular structure over the unit cell in Fig. 3 to draw out similarities and contrasts between the packing arrangements. Notably, in the γ-form crystal structure, the chains are no longer packed in rectangular blocks along the long-axis (lamellae) as in the *n*-alkane structures but instead are offset in a ‘staircase’ between adjacent aligned chains. This arrangement produces a plane of hydrogen bonding inclined relative to the long-axis of the hydrocarbon chain. A further plane of van der Waals interacting methyl groups also appears between chain ends not terminated by the alcohol moiety. The β-form likewise exhibits this alternation between hydrogen bonded alcohol terminal groups and end-to-end methyl group planes. However, in the β-form the planes containing hydrogen bonding form very nearly perpendicularly to the long-axis of the molecular chain. *Gauche* conformations in every other alcohol group support this arrangement in contrast to the all-*trans* conformations in the γ-form (and following the all-*trans* conformations in the *n*-alkanes). The methyl terminal groups at the other chain end, nevertheless, remain in the all-*trans* conformation. This arrangement resembles a more block-like arrangement akin to the *n*-alkanes, and in fact the β-form has sometimes been assigned to an orthorhombic cell.^75^

The even-carbon *n*-carboxylic acids follow a similar pattern to the *n*-alkanols. They likewise exhibit two polymorphs, denoted B (monoclinic space group *P*2_1_/*a*) and E forms (monoclinic space group *P*2_1_/*a*),^84,90,91^ and these two polymorphs contain either a *gauche* conformation at the polar group chain end (B-form) or an all-*trans* conformation at the polar group terminus (E-form). In either case, the carboxylic acid terminal groups are paired to form a hydrogen bonding network between adjacent molecules. Both forms also preserve aligned chain packing following the PE subcell. However, the hydrogen bonding network in both B- and E-forms of the *n*-carboxylic acids is inclined relative to the long-axis of the hydrocarbon chain, a contrast with the β-form containing *gauche* conformations at the polar chain ends in the *n*-alkanols. Additional orthorhombic polytypes, produced through alternation of the inclination direction in a doubled repeat of the B and E forms have also been reported.^90^ Phase transitions at elevated temperatures produce additional changes to packing.^82,89^ Notably the E form is considered metastable, with the B form (with *gauche* conformations) the lowest energy form,^90^ in contrast to the preference for the all-*trans* chain conformations in the stable γ-form for the even-carbon *n*-alkanols^79^ and the crystal structures of the *n*-alkanes.

**Fig. 4:**
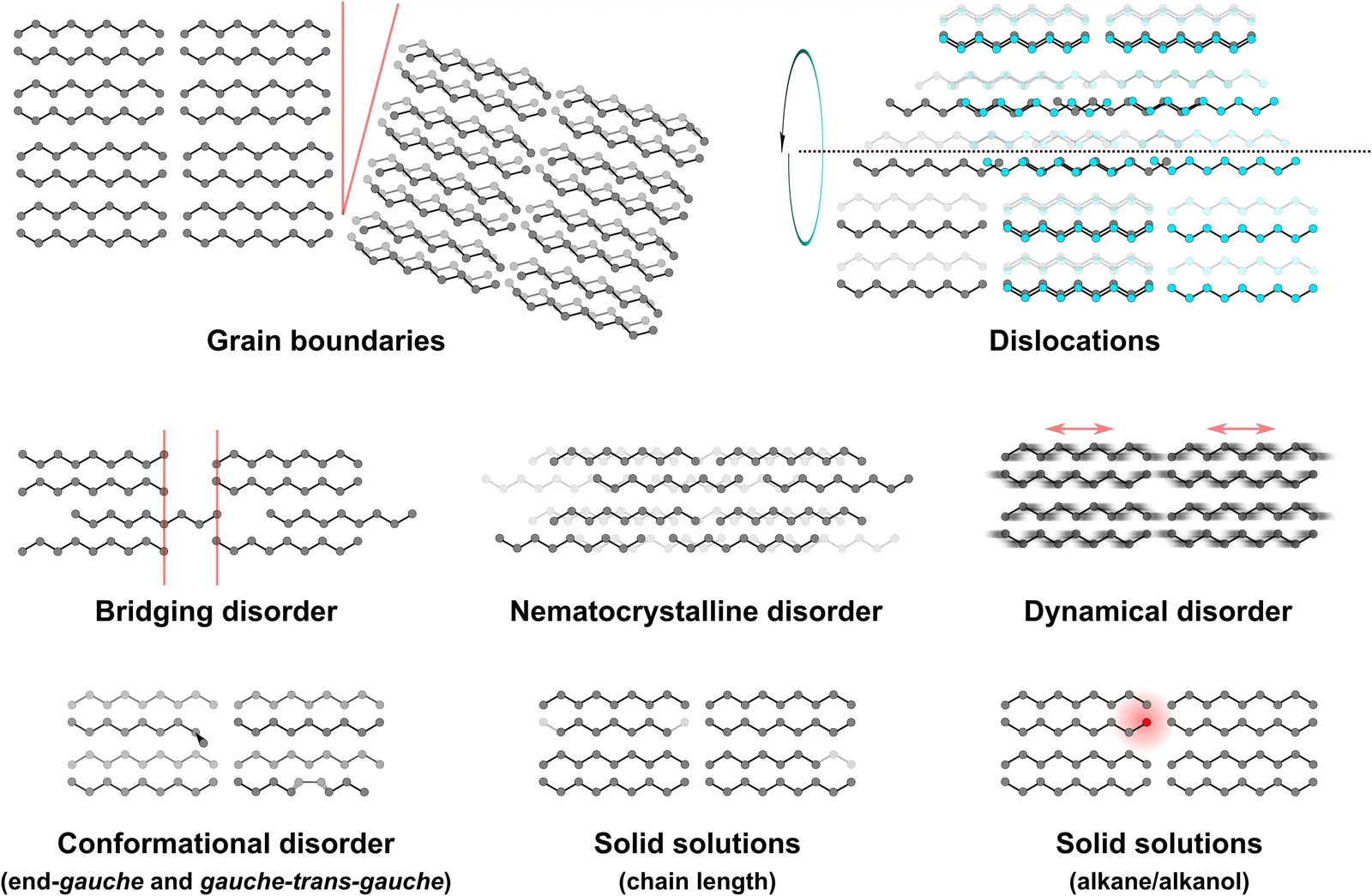
Illustration of the major disorder modes reported in *n*-alkanes and their solid solutions. An *n* = 9 (*n*-nonane) structure is shown for ease of visualisation to represent alkane structures relevant to leaf waxes generally which are generally hydrocarbons with *n* > 21.

The unit cell descriptions offer an important abstraction of the single-component, ordered molecular packing of waxes, outlined in Fig. 2 as parallel lines (blue regions). Whilst they do not offer insight into the structure of the disordered regions (red regions, Fig. 2), these unit cell descriptions offer an important reference point for a variety of disorder modes related to the ordered molecular packing. The use of the term ‘amorphous’ may apply to areas without any long-range ordering, but waxes are also known to exhibit a range of disorder types that very much comprise diffracting (*i.e.* crystalline) phases. These disordered phases are expected to introduce locally distinct functional properties as the atomic positions deviate from the crystalline ordering. Such displacements leave additional gaps for molecular diffusion (chemical properties) or altering the slip planes (and therefore mechanical properties), meaning the ‘bricks’ in the barrier-membrane model (Fig. 2) may themselves contribute to permeability. The presence of isoalkanes in predominantly linear-chain wax mixtures alter the fraction considered amorphous and in turn modify the storage modulus in dynamical mechanical measurements.^92^ A significant body of this work has arisen in this area from oil sector research on long-chain hydrocarbons where the formation of solids in liquid fuels presents key challenges. Nevertheless, the underlying structural features are highly relevant for leaf wax systems as similar features have been observed across chain lengths and compositions seen in plant waxes. These disorder modes, observed in single, few, and multi-component wax mixtures, are illustrated each in turn in Fig. 4.

The simplest source of structural heterogeneity is perhaps the grain boundary. These may exist in single-component polycrystalline materials at the juncture between single-crystal regions but will also appear in multi-component waxes (both between like or dissimilar grain compositions). A single-crystal region or grain in this sense implies an ordered region with a consistent orientation. If two adjacent crystals are at very different orientations, they may exhibit large angle grain boundaries; if they are at similar orientations they may exhibit small angle grain boundaries. A step or discontinuity of a crystal otherwise at a similar orientation may also suggest a grain boundary. Small angle grain boundaries have been observed in high-resolution electron microscopy^93^ as well as in scanning electron diffraction (SED) measurements^18^ of single-component waxes as well as binary mixtures. These grain boundaries have been attributed to growth and partial fusion of separately crystallising nuclei with similar but not identical orientations. Disordered regions between steps have also been observed in high resolution images of binary mixtures.^93^ Adjacent regions with different chain orientations (and therefore different crystal orientations) in *n*-alkanols, as detected by changes in adhesion signals in atomic force microscopy,^18^ further suggests particular grain microstructures can arise in lower symmetry waxes with polar groups. Scanning electron microscopy images of faceted epicuticular wax crystals (both on plant leaves and in mechanically isolated crystals)^10^ suggest that such grain boundary features are present in well-crystallised regions of leaf waxes.

A second defect often observed to be interlinked with growth of paraffin crystals is the dislocation. These are linear defects, defined by a local displacement (such as by insertion or deletion of a plane of molecules) in a single direction relative to a line in an otherwise ordered crystal. Where the displacement occurs along the line, the dislocation is said to be a screw dislocation as an additional plane creates spiralling steps in the crystal (Fig. 4); when the displacement is perpendicular to the line it is an edge dislocation, and at intermediate relative orientations the dislocation is said to be mixed. Screw dislocations have been studied in the growth of paraffin crystals, as evidenced by spiral growth morphologies.^94^ In turn, these have been modelled, indicating significant rotations occur in molecules near the dislocation line (*i.e.* the dislocation core)^95^ where the molecules are most displaced from the positions expected for defect-free packing. Edge dislocations have also been observed directly in lattice-resolution imaging down the chain axis.^15^ We have more recently developed low-dose SED techniques for analysis of curved mixed dislocations in *n*-hentriacontane (C_31_H_64_).^96^ As in PE, it is likely that partial dislocations and stacking faults may also occur together in PE-like wax crystals.^97^ Directly probing such defects presents a challenge within the cuticle, but may become tractable with advances in cryogenic electron microscopy sample. In the interim, atomistic modelling of such systems appears more feasible to probe their possible contribution to diffusion and mechanical properties in leaf waxes.

As conveyed already in Fig. 2b, chains can also span across otherwise discrete lamellae containing PE-like alignment within them. Here, we clarify that this feature can occur in single-component as well as wax mixtures, *i.e.* this feature does not inherently or exclusively arise from mixed chain lengths. Moreover, there are at least two crystallographically distinct modes of chains spanning adjacent lamellae: (i) bridging disorder may occur where the PE-like ordering is preserved almost identically on either side so as to match the interlamellar spacing to an integer repeat of the PE-chain (more precisely, a 3*c*_PE_/2 repeat, where *c*_PE_ is the *c*-axis of the PE subcell); (ii) alternatively, the chains may be disordered with no relationship to lamellar spacings in what is termed nematocrystalline disorder, following terminology used in describing liquid crystals with single common direction.^44^ The first case (i) will accordingly preserve reflections *hkℓ* of the types 00*ℓ* and 01*ℓ* whereas in the second case (ii) only PE-like reflections will be observed without any diffraction signature of lamellar ordering. These cases represent the extremes, and intermediate disorder will produce diffraction signals with varying relative intensities of the PE-like and lamellar reflections. Notably, the *n*-alkanol γ-form packing will also eliminate the visibility of lamellar ordering on *0kℓ*,^18^ and this arrangement can be seen as analogous to nematocrystalline ordering. The γ-form is ordered in principle, however, so multiple orientations (cuts through reciprocal space) or additional techniques are required to assess the degree of order in such systems.

Displacements along the long-axis of the chains also occur dynamically, *i.e.* as a result of thermal motion, rather than as static disorder. Dynamical disorder arising from thermal motion has a characteristic temperature dependence, and so these displacements have been evidenced by diffuse scattering in electron diffraction that can be linked to unidirectional sliding modes^18,98^ that is reduced at low temperatures in *n*-alkane crystals.^99^ This translational motion has likewise been observed in diffuse scattering in X-ray diffraction in long-chain *n*-alkanols, with an estimated motion up to 20% of the repeat period.^100^ Beyond whole-chain disorder, local conformational disorder has also been identified within chains. The single-crystal structures containing *gauche* terminal conformations already suggest a low energy barrier to such alternative conformations, and so these may occur as defects within all*-trans* crystalline ordering. Kinked chain models^99^ as well as the incorporation of branched alkanes^101^ have been put forward. Most notably, in mixtures with different chain lengths (*e.g.*, binary mixtures of alkanes), end-*gauche* defects, including in the form of dynamical conformational motion observed in nuclear magnetic resonance spectroscopy, occur in the longer chain component of the mixture (in addition to translational motion of the shorter chains) more prominently than in single-component solids.^102^

These observations on mixtures, as implied in Fig. 2b, outline the possibility of solid solution formation in multi-component mixtures. Whereas other disorder modes appear in both single-component and multi-component waxes (with varying contributions linked to composition), solid solution behaviour (as well as phase separation beyond a given fraction for limited solid solutions) arises directly from mixed compositions. Fig. 4 outlines two distinct classes, with mixed chain length and mixed functional group chemistry not presented as mutually exclusive features but as two conceptual groupings. Given the difference in single-component unit cells for *n*-alkanols and *n*-carboxylic acids (Fig. 3), their introduction in mixed waxes is expected to offer a different dimension to *n*-alkane mixtures. Consequently, many studies on the simplest binary mixtures have focused on binary solid solutions of either *n*-alkanes,^45,103–107^ of *n-*alkanols,^108^ or of fatty acids.^109^ A more limited number of studies have begun to unravel binary mixtures of *n*-alkanes and *n*-alkanols, either with matched number of carbons^110^ or deviating by one carbon.^18^ These studies follow from the principle established in *n*-alkane binary mixtures that only a certain molecular volume difference can be accommodated within a stable solid solution,^111–114^ typically within a few carbon atoms in the chain length. Symmetry rules for solid solutions (such as those arising from differences in odd- and even-carbon *n-*alkanes) appear to have minimal effect in *n*-alkanes.^115^ A volume difference that is too great will result in either the formation of a metastable solid solution that then phase separates or the formation of a eutectic microstructure directly from the melt, depending on the particular system.^104,111^ Such phase separation processes have also been observed in ternary mixtures.^105^

For binary mixtures, an average composition model with partial occupancies at the chain ends can offer a single unit cell description of such solid solutions, *e.g.* a C_34_H_70_ cell for a solid solution of C_32_H_66_ and C_36_H_74_.^116^ This approach can be used much more widely, for indexation of complex mixtures in petroleum-derived, synthetic, and natural waxes.^117,118^ The average composition model has been shown, however, to need refinement beyond a single average composition when applied to distributions of chain lengths. In particular, efforts have been made to distinguish normal logarithmic distributions (in Fischer–Tropsch waxes and solid deposits in diesel) and exponential decreasing distributions (typical in petroleum fluids) with the normal logarithmic distributions forming a single solid solution and exponential decreasing distributions forming multiple, distinct solution phases.^119^ Analyses of normally distributed waxes with much longer chains added show that only a minor fraction can be accommodated within a single solid solution.^120^ The distribution of chain lengths also plays a role in crystallisation, with longer chain *n-*alkanes offering nucleation sites for shorter chains.^121^ In plant leaf waxes, there is often a substantial similarity in the chain lengths of the major wax components, suggesting the feasibility of stable solid solution formation. Still, minor components and the role of mixed functional group chemistry in complex mixtures remains an area of active investigation as these may phase-separate despite similarity in chain lengths depending on the relative composition.

In the context of leaf waxes, a further aspect worth noting is the effect of the local environment on crystallisation (such as effects of interfaces and surfaces across the cuticle). In isolated waxes, the role of surface templating is well-documented with orientation-controlled, templated crystallisation demonstrated on benzoic acid,^93^ naphthalene,^122^ polyethyene,^123^ and graphite.^124^ Other surfaces have also shown to have a pronounced effect on wax crystal formation, including on surfaces of fatty acids and glycerols^125^ and on ice.^126^ Simulations likewise indicate significant ordering of linear chain solvents as well as poly(octadecyl acrylate) polymers at wax surfaces.^127^ Within leaf waxes, this raises an important question on whether wax layers control the addition of further wax deposits during increases in wax content at surfaces or whether cutin, polysaccharides, or other matrix components direct or template the formation of leaf waxes. Surface and interface effects also introduce a further point of consideration in applying observations from isolated and, especially, recrystallised waxes directly as models for leaf waxes. That is, care must be taken to assess the effects of recrystallisation conditions (including surfaces or other templating compounds) as these may introduce texture (preferred orientation) or modify the distribution of defects, disorder, and grain microstructure. These structural reference points, nevertheless, provide a critical basis for understanding the types of order and disorder that are present in waxes corresponding to leaf wax compositions (providing crystallographic descriptors for the qualitative picture represented in Fig. 2) and also in identifying thermodynamic features pertaining to solution stability and phase separation which may begin to elucidate how and why leaf waxes exhibit their defined composition and chain length distributions.

**Fig. 5:**
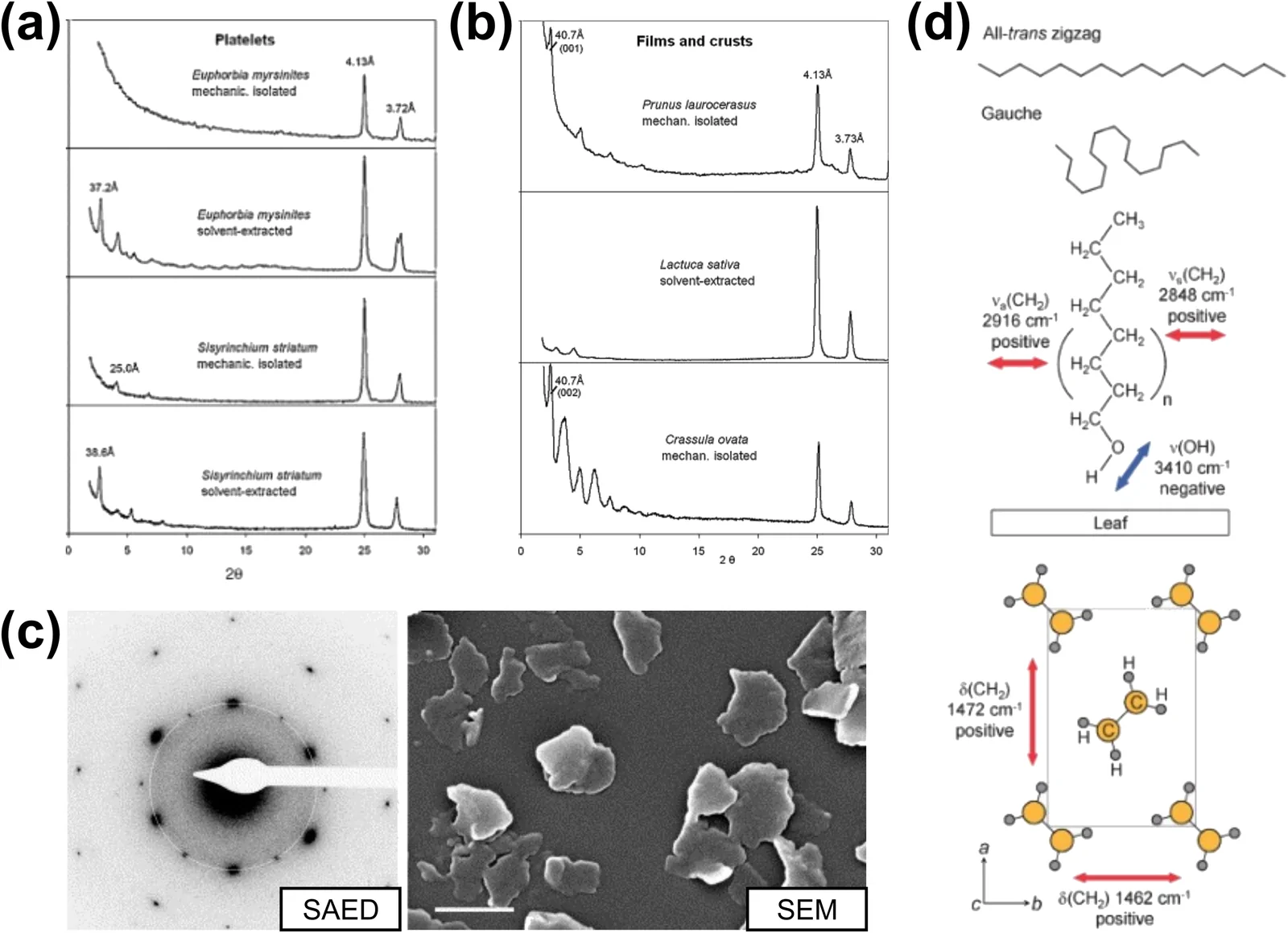
X-ray diffraction acquired on (a) platelets and (b) films of epicuticular waxes. (c) Selected area electron diffraction (SAED) and SEM imaging of platelets from *Yucca filamentosa.* (d) Schematic illustrating the interpretation of polarization modulation-infrared reflection–absorption spectroscopy (PM-IRRAS). Panels (a)–(c) are reproduced from Ensikat *et al.*^10^ with permission (copyright 2006, Elsevier Ireland Ltd) and panel (d) is reproduced from Hama *et al.*^130^ with permission (copyright 2019, the authors).

We conclude this section by returning to the leaf itself and reviewing the state of knowledge on the structural organisation of waxes in the epicuticular and intracuticular waxes. Epicuticular waxes offer the most straightforward comparison, as these exhibit X-ray and electron diffraction in mechanically isolated plates, films, and tubules.^10^Fig. 5a and b highlights a range of examples of powder X-ray diffraction patterns obtained on both mechanically isolated and solvent-extracted and re-crystallised epicuticular waxes. Almost all patterns show strong intensity from two peaks at 25–30° (2*θ*, Co Kα radiation with a wavelength corresponding to 1.79 Å).^10^ These features correspond to characteristic spacings of an orthorhombic PE-like cell or subcell and are the same reflections observed in selected area diffraction (Fig. 5b) for PE-like crystals viewed along the long-axis of the chains (Fig. 3 top panels). Notably, differences appear in the low-angle reflections with some epicuticular waxes showing a series of intensities less than approximately 10° (2*θ*) typical of 00*ℓ* spacings and ordered end-to-end packing. Alternating intensities (Fig. 5b, *C. ovata* or jade plant) are consistent with double-layer packing characteristic of the β-form of *n*-alkanols in some leaf waxes, a feature also indicated by step-height analysis of isolated plates from *Yucca aloifolia* indicating 7–10 nm steps^128^ corresponding to double-chain step heights expected for common chain lengths in leaf waxes.^18^ Single (*ca.* 4 nm) and double-chain (*ca.* 8 nm) steps have been observed in AFM of recrystallised waxes.^129^

Differences also appear between solvent-extracted and recrystallised waxes and mechanically isolated waxes, suggesting the recrystallisation can increase crystalline ordering over what is present natively on the leaf. Corroborating observations of all-*trans* conformations in orthorhombic or monoclinic packing (*Brassica oleracea* L. and *Kalanchoe pinnata*) have been made using infrared (IR) spectroscopy, particularly using polarised IR (Fig. 5d) to also reveal the near-perpendicular orientation of chains to the leaf surface.^130,131^ These observations pose an important further question on the role of preferred orientation effects in X-ray and electron diffraction observations as plate-like and other anisotropic structures will often exhibit significant modification of observed intensities in powder X-ray diffraction data and present particular zone axis orientations in electron diffraction. The observation of *ca.* 100 nm scale ordered domains exhibiting 00*ℓ* reflections in spatially resolved nanobeam scanning electron diffraction of pure replica waxes^18^ suggests powder X-ray diffraction may also struggle to resolve nanoscale ordering in epicuticular or intracuticular waxes. The occurrence of ordered domains in replica waxes also decreases with increasing *n*-alkanol content,^18^ suggesting that the particular compositions present in leaf waxes may contribute to the presence or absence of lamellar ordered structures.

AFM-IR studies of *Sorghum bicolor* likewise confirm variation in the degree of crystalline order across the epicuticular wax.^132^ Moreover, methylene stretching frequencies indicate *gauche* conformations in addition to all-*trans* arrangements.^132^ Intense methylene signatures at wax crystal edges has been attributed to templated growth,^132^ in line with the role of interfacial crystallisation in isolated waxes. Spatial heterogeneity in carbonyl and methylene signatures^132^ may also suggest chemical phase separation occurs on these leaf surfaces.

Less has been documented to date on the crystalline order or disorder in intracuticular waxes without recrystallisation. On a larger scale, Raman spectromicroscopy has been used to map out the distribution of long-chain aliphatics, cutin, calcium oxalate, and polysaccharides in *Picea abies* (Norway spruce) needles.^17^ These results confirm a highly ordered epicuticular wax (such as indicated by sharp bands at 1120 cm^−1^) with significant greater disorder indicated by the less sharp features in the underlying layers^17^ (the location of intracuticular waxes). However, there is significant spatial overlap in the sharp (crystalline) features as analysed by non-negative matrix factorisation^17^ suggesting a possible mixed ordered/disordered composite structure in the intracuticular waxes. TEM studies of leaf cross-sections spanning the epicuticular wax to the cell wall using resin embedding and staining techniques have been reported, with some indications of what is referred to as a lamellate structure in the cuticle proper just below the epicuticular wax layer.^25,133–135^ This lamellate structure should be distinguished clearly from the lamellae of the *n*-alkane unit cells as it occurs on a much larger length scale. Striations have been attributed to wax-enriched regions^136,137^ which disappear after wax extraction,^138^ suggesting there may be multi-scale or hierarchical order of waxes within the cuticle.

Overall, bringing these on-leaf and isolated and replica wax analyses together serves to highlight correspondence between the crystalline features in epicuticular waxes, particularly the average structures represented in the unit cell descriptions. It also serves to identify the knowledge gaps between the microstructural variety of disorder modes in simplified models, the phase diagrams of wax mixtures, and which features are present in leaf cuticles across varied plant species. The ordering within wax-rich regions within the cuticle (*i.e.* intracuticular waxes) remains a largely unresolved question by direct methods of characterisation, relying at present on inferences from simplified and isolated waxes. This question is significant as the intracuticular waxes dominate many of the defining chemical and mechanical properties of cuticular membranes, which we turn to next.

## 4. Route 2: leaf waxes as diffusion barriers

In contrast to the preceding section which traces a route building up structural descriptions of waxes from simplified and often isolated and recrystallised wax components (the upper arrow in Fig. 1), we now turn to research focused on the properties of leaf waxes and the cuticular function. This represents the second path (lower arrow in Fig. 1) focused on bulk analyses leading toward the common objective for identifying underpinning (and microscopic) structure–function relationships. We focus this section of the review on three key questions: (1) how do leaf waxes interact with water in creating a transpiration barrier? (2) How do agrochemical active ingredients (AIs) and adjuvants interact with leaf waxes? And (3) which components of the leaf cuticle (and the waxes they contain) determine these properties?

The permeability of the leaf cuticle to water has been studied extensively, with seminal work on enzymatically isolated cuticular membranes establishing key gravimetric methods of analysis.^139^ These methods have remained relatively unaltered in over 40 years (ref. 140) though they have also been benchmarked against ^3^H-labelled water approaches.^6,141,142^ The gravimetric method involves making a water-tight seal on the cuticle to a container filled with water which is stored upside down over a drying agent (*e.g.* silica gel) and monitored gravimetrically to determine the permeance.^139,140^ Values spanning 10^−11^ to 10^−9^ m s^−1^ have been recorded for leaf cuticles with variation ascribed to environmental adaptations across >24 species, including leaves popular among these and later analyses from *Citrus aurantium* L. (bitter orange), *Hedera helix* (ivy), *Lycopersicon esculentum* (tomato), and trees *Prunus laurocerasus* L. (cherry laurel), *Ginkgo biloba*, and *Juglans regia* L. (English walnut).^143,144^ Decreases in permeance on storage have been interpreted as reflecting dynamical changes in wax crystallites upon drying^143^ also suggested in humidity-dependent studies indicating increased permeability correlated with humidity in leaf disks and isolated cuticular membranes which have been used to suggest polar pores as distinct from a non-polar path through the cuticle.^141^ This work has raised the question on the role of polymeric cutin in providing free carboxylic acid groups in polar and ionic species transport across the cuticle.^141^

Work has over time turned attention more closely to which of the epicuticular or intracuticular waxes create the primary transpiration barrier. Vogg *et al.* used a combined approach analysing intact cuticles with sequential removal of epicuticular waxes with gum Arabic and intracuticular waxes with chloroform as well as genetic mutations (*LeCER6*) to modify wax composition on tomato leaves and fruit (characteristically dominated by hentriacontane, C_31_H_64_ in the wild type); this analysis identified the very long chain aliphatics (VLCAs) in the intracuticular wax as dominating the transpiration barrier, with smaller effects from alicyclic compounds and epicuticular wax.^145^ These findings have been supported by the work of Jetter and Riederer in selecting VLCA-rich (*e.g. Schefflera elegantissima*) and alicylic-rich species (*e.g. Citrus aurantium*) for study.^5^ Here, the dominant role of VLCAs in the VLCA-rich intracuticular wax as well as the dominant role of the VLCA-containing epicuticular wax in the species with alicylic-rich intracuticular waxes has been established.^5^ Altogether, this work points to a major role of the VLCA component, with no correlation between the permeance and the total wax or wax thickness.^5^ Work across other species (including the frequently studied *Prunus laurocerasus* L., *Clivia miniata,* and *Hedera helix*) using sequential removal and extraction approaches has further confirmed the predominant contribution to the transpiration barrier by intracuticular rather than epicuticular waxes^6,142^ and that triterpenoid extraction makes minimal difference to transpiration in comparison with strong effects from VLCA extraction.^140^

**Fig. 6:**
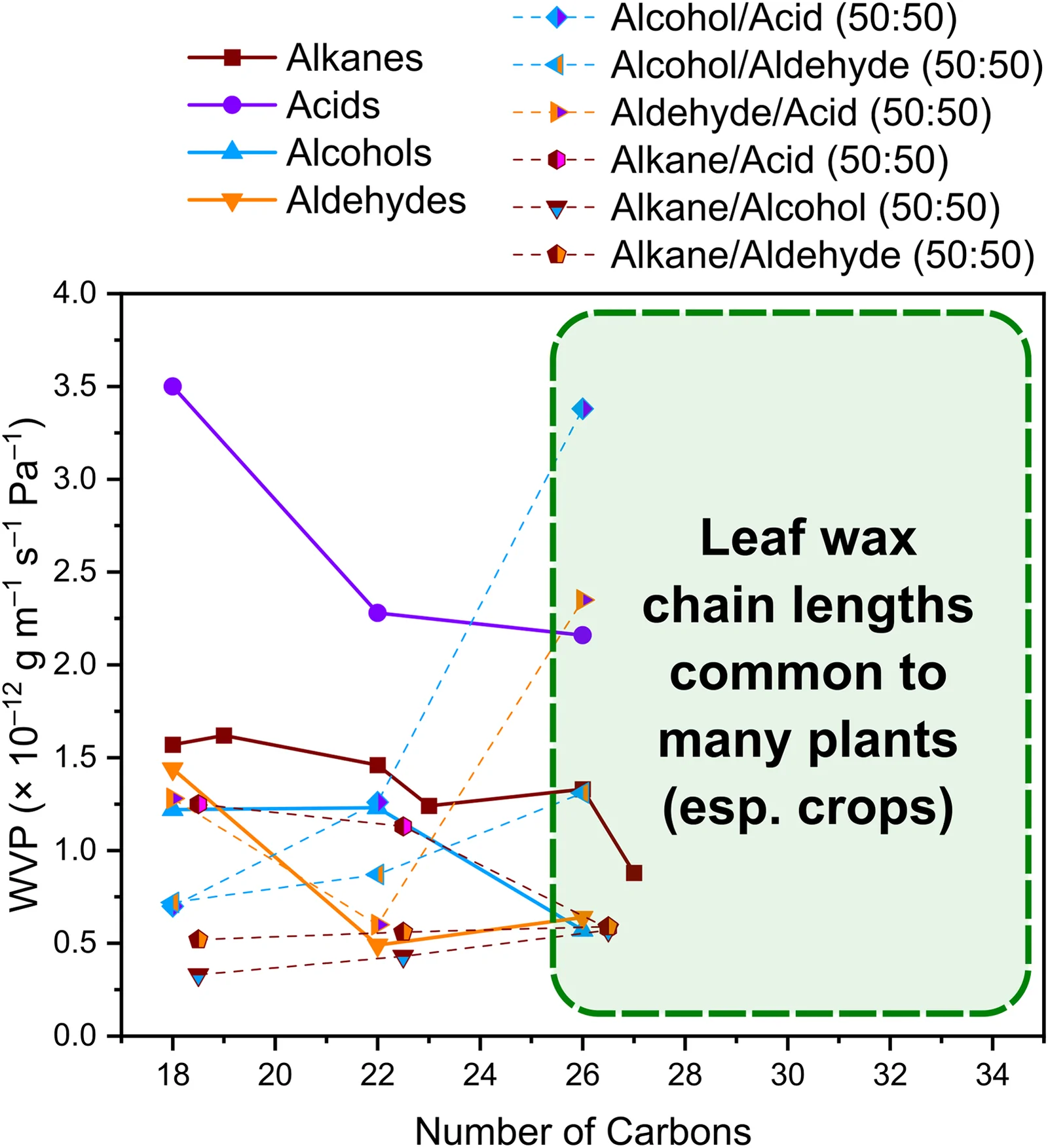
Average water vapor permeability (WVP, 23 °C, 100–0 RH% gradient) of model VLCAs and binary mixtures (50 : 50).^148^ For mixtures with different chain lengths, the number of carbons is plotted as the average. Expanding on these results and linking these properties to the microstructural structures present across more chain lengths and composition ratios will unlock structure–function relationships for leaf waxes.

In parallel, work on simplified, model waxes (*e.g.* C_22_H_45_OH/C_32_H_66_ modelled on *Clivia miniata* Regel, comprising 68% alcohols and 16% alkanes)^146,147^ and reconstituted waxes (*e.g. Triticum auestivum* L. (wheat), comprising ∼66% alcohols)^57^ have begun to elucidate some of the underlying mechanisms of interaction between water and VLCAs, at least in alcohol-rich wax compositions. Water significantly reduce transition enthalpies (by differential scanning calorimetry) in hydrated model waxes relative to their dry forms, suggesting water increases disorder, a possible plasticising effect, possibly through hydrate-formation with alcohol functional groups.^146^ Quartz crystal microbalance with dissipation (QCM-D) studies have likewise indicated a softening in the mechanical properties on water exposure.^147^ Neutron reflectivity and spectroscopic ellipsometry studies point to significant swelling of such waxes, with up to 50% of reconstituted wheat wax films made up of water (reversible on drying).^57^ The reversibility may arise from reformation of the original structural order on drying, or may point to uptake on surfaces and between crystallites. In a comprehensive study of single-component and binary systems, Leyva-Gutierrez and Wang have analysed variation in water vapor permeance (WVP) alkanes, alcohols, aldehydes, and fatty acids.^148^ WVP is highest among the fatty acids (associated with *gauche* conformations and favourable spacings for hydrogen bonding with water), whereas alcohols in pure form exhibit lower WVP than alkanes.^148^ In binary mixtures, however, alcohols introduce greater structural disorder (reduced crystallinity).^148^ Crystalline PE-like chains are understood to be a driving feature for limiting WVP.^148^Fig. 6 presents the WVP values for these pure compounds and binary mixtures (tabulated in ref. 148), highlighting the relevance to chain lengths in leaf waxes and especially crop leaf waxes (albeit also an area for further investigation to correspond to the predominant chain lengths and appropriate mixture fractions in leaf waxes).

The transport of agrochemical active ingredients and the role of adjuvants (compounds assisting their delivery) has likewise formed a parallel strand of research, focusing conceptually on the lipophilic or nonpolar path though ultimately drawing on many similar principles to the transpiration of water. Early methods have focused on loading isolated cuticular membranes and reconstituted leaf waxes with ^14^C-labelled compounds to use desorption measurements to establish diffusion coefficients, permeance, and cuticle/water and wax/water partition coefficients.^149,150^ More recently, loading-desorption analyses by liquid chromatography-mass spectrometry^151,152^ and adsorption-diffusion across wax films by attenuated total reflectance Fourier transform infrared spectroscopy have presented alternatives to radiolabelling.^16^ The role of adjuvants as accelerants (*i.e.* increasing mobility of the AI) has been established early, with models outlining path length (tortuosity) and size selectivity (free volume in the wax) as key parameters.^150,153^ Species tested have covered a similar breadth as in water studies, including *Citrus aurantium* L. (bitter orange), *Lycopersicon esculentum* L. (tomato), *Prunus laurocerasus*, *Ginkgo biloba*, and *Juglans regia* as well as barley leaf waxes (*Hordeum vulgare).*^154^ Compounds modelling active ingredients have included 2,4-dichlorophenoxy acetic acid (2,4-d), salicylic acid, benzoic acid, metribuzin, 4-nitrophenol, atrazine, pentachlorophenol (PCP), triadimenal, bitertanol, tebuconazole, and bifenox and adjuvant models have included a range of mono- and polydisperse alcohol ethoxylates (AEs) and alkyl phosphates.^52,149,150,153–158^ The analyses of model active ingredients have themselves pointed to differences in plant adaptations to their environment, with more drought-resistant species exhibiting higher barriers.^153^ The general interpretation has been that, in replicating behaviour in reconstituted waxes, lipophilic compounds diffuse through amorphous wax with the tortuosity defined by the arrangement of crystalline PE-like wax domains.^153^

A recurring question has been the role of the adjuvants, particularly whether they serve to solubilise the active ingredients on the leaf surface only, whether they penetrate the cuticle or wax, whether they act as plasticisers, alter the wax structure irreversibly, or remove (dissolve) leaf waxes. Adjuvants must work in a subtle and selective way: an effective adjuvant cannot work in a way that causes water loss to the plant; the role of the adjuvant must be selective for the active ingredient against loss of water. Different adjuvants have been used to establish distinct roles. For example, an AE (C_12_E_8_) was shown to increase the mobility of PCP in reconstituted barley waxes by a factor of four, ascribed to an unspecific plasticising effect that is reversible and, as such, not causing the destruction of crystalline wax domains.^155^ The effect of AEs show effects that vary on the size of active ingredients, indicating they contribute to increasing the free volume for diffusion.^156^*N*-alkyl esters have shown greater non-specific plasticisation, suggesting chemical interactions are critical for uptake or penetration in amorphous wax regions.^154^ Notably, in analyses of isolated cuticular membranes rather than reconstituted waxes, the plasticising effect of tributyl phosphate has been ascribed also to interactions with polymeric regions (cutin) as a way to explain the effect's reversibility.^157^ DSC analyses and local thermal analyses on surfactants like Synperonic A7 and A20 have shown plasticisation behaviour (in the form of melting temperature depression) for A7 but not for A20, indicating chemical variation in uptake or penetration by intact leaves and reconstituted waxes.^159^

The diffusion of other organic compounds (*e.g.* phenanthrene, a model pollutant) have been observed directly by two-photon excitation microscopy on maize (*Zea mays*) and spinach (*Spinacia oleracea*) leaves and in model waxes (octacosane C_28_H_58_ and paraffin waxes containing a distribution of chain lengths).^160^ Notably, these observations confirm the penetration of molecules into waxes, with clustering and inhomogeneity appearing in leaves and paraffin wax mixtures, suggesting chain length distributions likely create preferential areas for diffusion.^160^ Similar analyses on intact leaves have been ascribed to interactions with polymeric lipids rather than solid waxes in the leaf.^161^ However, sequential removal approaches in isolated cuticles of *Garcinia xanthochymus* (97% VLCAs, predominantly tritriacontane and hentriacontane) and *Prunus laurocerasus* (88% alicyclic, VLCA content dominated by nonacosane and hentriacontane), following similar approaches used for evaluating the active fraction in determining the water transpiration barrier, indicate VLCAs likewise contribute to the barrier for organic solutes (lipophilic and hydrophilic) with alicylic compounds contributing negligibly.^152^

Irreversible softening of waxes has also been observed for surfactants (hepta (ethylene glycol) mono iso-decyl ether and 2-ethyl hexyl glycoside), explained by adsorption and entrapment between wax grains.^147^ Studies evaluating the effects of penetration as well as spreading on the leaf surface, point to the dominating contribution of adjuvant acceleration through penetration of the leaf cuticle.^151^ Notably, concentration dependent studies on reconstituted wheat leaf wax have suggested a changeover in mechanism between physically reversible penetration of wax films below critical micelle concentrations (*e.g.* for glycol monododecyl ether, C_12_E_6_) and wax removal (dissolution) above the critical micelle concentration.^162^ This changeover has also been observed by exposure of intact barley leaves with tetraethylene glycol monododecyl ether with reversible effects observed at low coverage (and recovery of visible wax platelets) but significant increases in transpiration and wax loss at high coverage treatments.^163^ These findings underscore that the relative amounts of adjuvant and wax (concentration) is a controlling feature for the interaction.

Cumulatively, these studies indicate a significant role of long-chain waxes in determining the diffusion of water out and organic species into plant leaves. These interactions are complex due to the composite cutin and wax structure of the cuticle and vary between species as well as active ingredient and adjuvant chemistry. Plasticising effects (from water or adjuvants) also invite further study of different disordered or amorphous structures formed to further refine this putative mode of action. Triterpenoids and other alicylic compounds do not at present appear to play a major role in water or active ingredient diffusion phenomena, but have been identified in determining mechanical properties and thermal stability.^164,165^ More generally, wax mechanical properties change on sequential extraction from isolated cuticular membranes (as used in transport barrier studies),^166^ pointing to the multi-functional role of these bio-materials.

## 5. Unresolved questions and future directions

In this review, we have highlighted significant advances both in understanding the role of cuticular components in regulating water and organic molecule diffusion in plant leaves as well as in understanding the structures and disorder modes of pure and multi-component waxes. Yet there remains a significant gap in linking up these research directions. Detailed mechanistic models for the pathway for diffusing species, whether they interact with the cutin or wax domains in the leaf, and how they move along wax surfaces and grain boundaries or through disordered regions and amorphous waxes requires analyses of molecular packing that are matched and validated (for simplified wax models) and correlated definitively to key structures in the complex system of the leaf cuticle.

Connecting crystallographic probes for simplified leaf wax studies, analysis of reconstituted leaf waxes, and eventually direct, microscopic analyses of isolated cuticles and leaves are critical for further development. Multimodal approaches are very much needed where they are feasible, including studies on the texture and orientation of waxes, their phase fraction, and their spatial distribution of crystalline and amorphous regions in leaves. We believe these may become tractable in the near future building on the extant advances in suitably low-dose electron diffraction that now offer nanoscale resolution^18^ coupled with techniques in cryogenic electron microscopy and focused ion beam preparation of cross-sections of hydrated biological specimens. Emerging characterisation approaches also in THz spectroscopy of leaf waxes^167,168^ may open new avenues for tracking water and structural changes in the cuticle given the high sensitivity of THz spectroscopy to water and structural disorder. These spectroscopic studies, alongside infrared and Raman as well as NMR spectroscopies, have the potential to refine understanding of the molecular arrangements in the amorphous as well as crystalline phases. Pair distribution function analyses,^169,170^ including those possible with nanometre resolution using electron scattering,^171–173^ may also provide a means to link structural and spectroscopic analyses of these complex phase mixtures.

Parallel routes toward increasing complexity from studies of simplified waxes lie in developing improved phase diagrams and models for multi-component distributions of chain lengths. We see the common structural motifs in wax crystals of varied compositions as well as common disorder modes that provide means for intermixing many different wax chains within partially ordered crystals as cause for optimism in building on the central concepts of the barrier membrane model, where the presence of regions of low molecular mobility dominate the tortuosity-defined barrier to diffusion. At the same time, there is still a lot of detail to be worked out in linking subtle variations in composition and between species and environmental response to water diffusion and adjuvant interactions. WVP studies on single and binary wax compositions are promising,^148^ but remain some distance from both detailed crystallographic models and the compositional complexity in leaf waxes where even the simplest may contain three or four major components. Progress may be particularly tractable in major crop plants that have only a limited number of major components governing structure and function. Here, rigorous microstructural wax models will point to specific targets for controlling diffusion pathways through the leaf cuticle for crop protection.

We believe there are several specific directions for future research where bridging these gaps will make significant impact. In the development of crop protection products, precise and experimentally informed parameters for models and simulations of transcuticular uptake can be refined from improved multi-scale (nm-to-mm) characterisation of leaf and replica waxes. For example, the development of digital twins for simulating the tortuosity through lamellar, disorder, and amorphous wax domains or cutin-wax composites together with the use of experimental structural data (with associated functional response measurements) to define modelling parameters like mobilities, pathlengths, and partition coefficients^150,157,174^ as well as in modelling of the effects of droplet spreading and evaporation for active ingredient delivery.^175–179^ We also see benefits in understanding the biology of the leaf cuticle, by bringing structural studies together with rapidly expanding genetic techniques for probing and modifying wax transport, deposition and biosynthesis,^180–182^ total wax levels (especially *n*-alkane levels in drought conditions),^183^ or wax composition changes for drought tolerance.^184^ Deliberately tuning plant performance parameters by controlling their microscopic, crystallographic, and morphological characteristics, and the resulting transpiration barrier effects, would accelerate trait development. Focusing plant development on beneficial surface properties, *i.e.* based on sound scientific modification of the leaf to limit water loss without impacting photosynthesis or mechanical properties, may reduce the need for expensive whole field performance trials. Such approaches will also open routes for novel trait development across photosynthesis, frost resistance, and pathogen attachment and beyond leaves to include applications in seeds, flowers and shoots. More broadly, we believe these advances will elucidate needs for resilient plants, sustaining not only crop plants but wider ecologically important biodiversity in future.

## Conflicts of interest

The authors declare that they have no competing interest in this work.

## Data Availability

No primary research results, software or code have been included, and no new data were generated or analysed as part of this review.
